# Challenges of Treating a C2 Odontoid Fracture in an Elderly Patient With Multiple Comorbidities: A Case Report

**DOI:** 10.7759/cureus.75242

**Published:** 2024-12-06

**Authors:** Davis A Melin, Ethan D Rich, Stephen J Despins

**Affiliations:** 1 Department of Osteopathic Manipulative Medicine, Liberty University College of Osteopathic Medicine, Lynchburg, USA; 2 Department of Surgery and Specialty Care, Liberty University College of Osteopathic Medicine, Lynchburg, USA; 3 Department of Sports Medicine and Physical Medicine, and Rehabilitation, Collaborative Health Specialty Services, Lynchburg, USA

**Keywords:** cervical orthosis, comorbid conditions, fracture pain management, geriatric fracture, odontoid process fracture, orthopedic sports medicine, physical medicine & rehabilitation

## Abstract

An 88-year-old male with a history of cervical spondylosis (status post laminectomy of C2-C3 and laminoplasty of C4-C5), chronic congestive heart failure (CHF), pulmonary embolism, and lumbar spinal stenosis presented to an outpatient sports medicine clinic with neck pain following a fall five days prior due to loss of balance. He reported pain on the left side worsened by movement and accompanied by neck “clicking.” A physical exam showed severe limitation in cervical spine extension limited by pain and loss of lordotic curve and a neurologic exam demonstrated weakness in the left leg secondary to a previous back surgery. A cervical spine X-ray revealed multilevel degenerative changes without evidence of fracture. To rule out a fracture, computed tomography (CT) was completed and revealed a new fracture at the odontoid process and the C2 right pars interarticularis. The consulting orthopedist recommended operative management due to the risk of atlantoaxial instability. Unfortunately, the patient experienced an acute episode of atrial fibrillation that worsened his CHF. With an overall heavy burden of medical comorbidities, the patient chose to receive hospice care and is being managed non-operatively. Type II odontoid fractures in the geriatric population require a complex risk/benefit analysis necessitating a collaborative approach in support of the patient’s health goals.

## Introduction

Odontoid fractures are associated with high morbidity and mortality, and they comprise 15% of cervical fractures in patients older than 65 years old [1]. Classically, there are three odontoid fracture patterns that are important for determining management. Type I is an oblique fracture through the odontoid tip; Type II is a fracture through the waist of the odontoid peg; Type III is a fracture inferior to the odontoid waist and into the body of C2 [2]. Typically, Types I and III fractures are treated non-operatively due to a high osseous consolidation rate [1]. Conversely, Type II fracture treatment is debated at length in the literature [3]. Type II fractures have limited healing potential due to the tenuous blood supply at the waist of the odontoid peg, along with poor bone quality and altered regional biomechanics [3].

For elderly patients with medical comorbidities, decisions concerning fracture management can be difficult because these fractures are associated with high mortality rates with both non-operative and operative treatment [3]. Non-operative treatment options for type II odontoid fractures include orthoses such as a hard collar or semi-rigid collar and halo thoracic vest, which have all been shown to be effective in facilitating bony consolidation in patients with non-displaced C2 fractures [1]. Operative treatments include odontoid screw fixation (anterior approach) and C1-C2 arthrodesis with instrumentation (posterior approach) [3]. In either treatment pathway, pain management and rehabilitation are key for maintaining function and improving quality of life [4].

This study aims to discuss the complexity of developing a treatment plan for a Type II odontoid fracture in an elderly patient with multiple comorbidities in the context of current evidence for best practice.

## Case presentation

Case history and physical exam

An 88-year-old male with a history of cervical spondylosis (status post laminectomy of C2-C3 and laminoplasty of C4-C5), lumbar spinal stenosis, osteoarthritis of multiple joints, chronic congestive heart failure (CHF), pulmonary embolism, major depressive disorder, epilepsy, and frailty presented to an outpatient sports medicine clinic with neck pain following a fall five days prior where the patient lost his balance and fell onto his hands and knees. This was his third fall in the last few weeks. He reported neck pain on the left side worse with movement and accompanied by neck “clicking.” Additional complaints included chronic bilateral paresthesias throughout his hands, fine motor control impairment in his fingers making it difficult to button his shirts, and left leg weakness secondary to previous back surgery. His current medications are listed in Table 1.

**Table 1:** The patient's current medications

Medication	Dose, route, frequency	Purpose
Bupropion	300mg, by mouth, daily	Major depressivedisorder
Furosemide	20mg, by mouth, every other day	Congestive heart failure
Propranolol	20mg, by mouth, daily	Congestive heart failure
Apixaban	4mg, by mouth, twice per day	History of pulmonary embolism and deep vein thrombosis
Phenytoin	100mg, 3 capsules by mouth, daily	Epilepsy
Indomethacin	25mg, by mouth, three times per day as needed	Pain secondary to osteoarthritis

Family history included his dad dying at age 52 from a cerebrovascular accident and having a history of emphysema, and his mom died at age 74 from heart disease and had a history of chronic obstructive pulmonary disease. The patient has a 20-pack-year smoking history and quit at age 40.

On examination, the patient's vital signs were a blood pressure of 115/70 mmHg, a pulse of 79 beats per minute, an oxygen saturation of 96% on room air, and a body mass index of 20.2 kg/m². The patient ambulated with a rolling walker and exhibited poor seated posture with difficulty keeping his head vertical. Active and passive range of motion testing revealed severe limitation in cervical spine extension likely due to pain and loss of the cervical lordotic curve. Tenderness was present at C4 to C6 along the left paracervical musculature. Compared to previous visits where the patient was managed for other musculoskeletal complaints, strength, and sensation in the bilateral upper and lower extremities were unchanged, with weakness present in the left leg. Reflexes were symmetric and normal in the bilateral upper and lower extremities, and the Hoffman sign was negative bilaterally.

Differential diagnosis

Considering the patient's history and physical exam findings, the following diagnoses were considered: cervical spine fracture, cervical strain/sprain, cervical radiculopathy, cervical myelopathy, cervical stenosis, cervical somatic dysfunction, cervical trigger point, cervical internal disk disruption, and cervical facet joint-mediated pain. 

Tests and results

**Figure 1:**
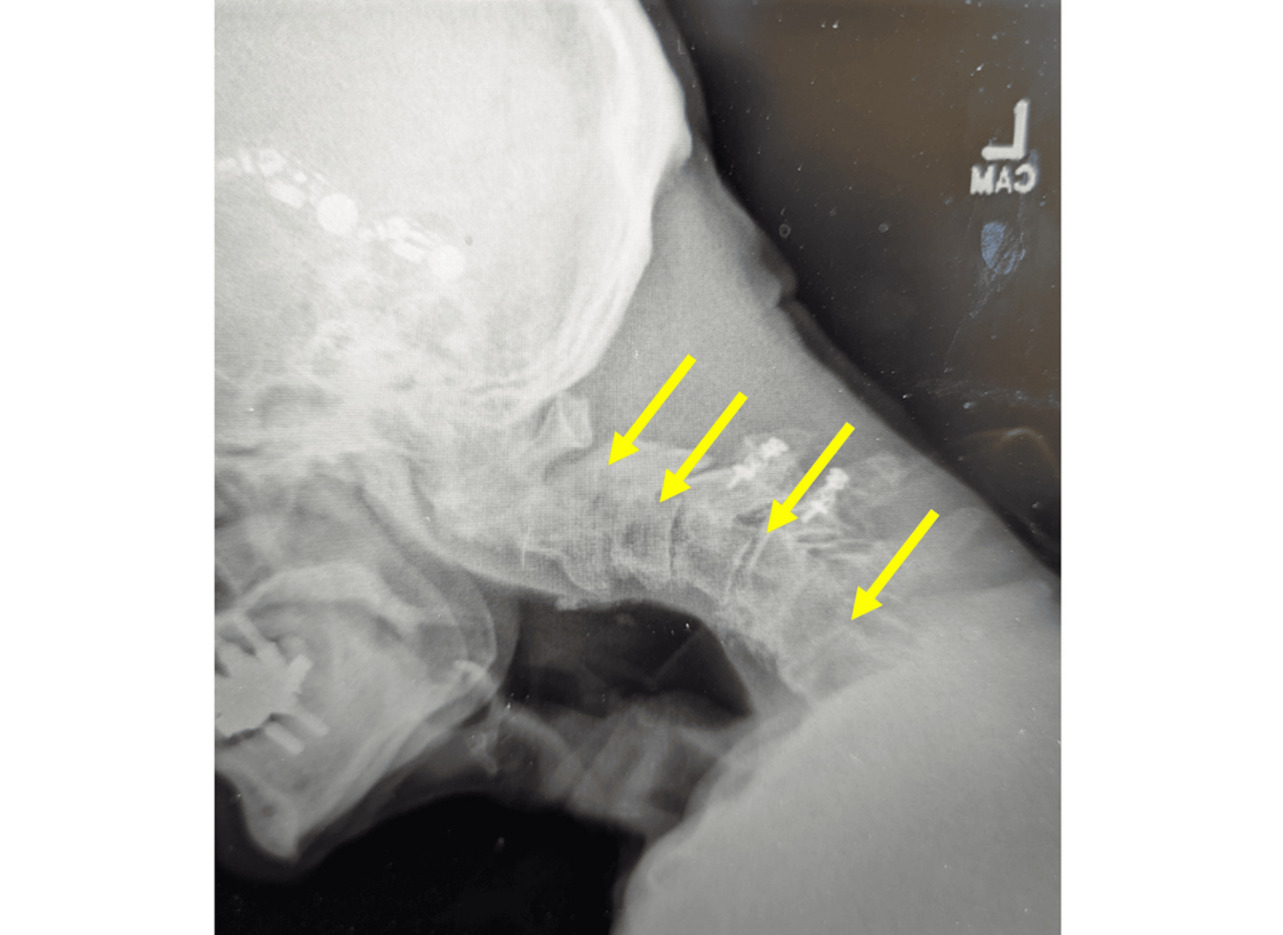
Cervical X-ray findings revealed multilevel degenerative changes (indicated by yellow arrows) without evidence of fracture.

The cervical spine X-ray was negative for fracture (Figure 1).

**Figure 2:**
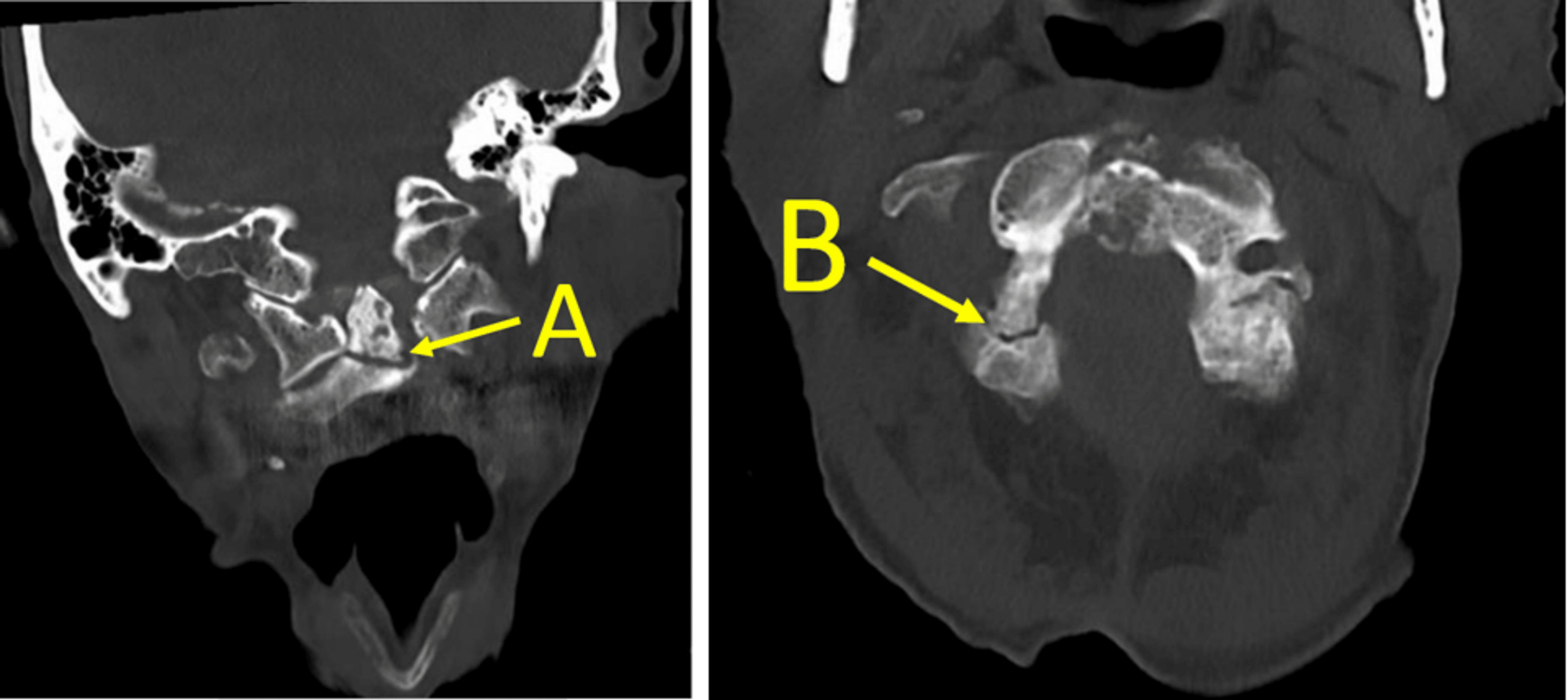
New fracture at the odontoid process (Type II)(image A) and the right C2 pars interarticularis (image B) with a grade 1, 7mm anterolisthesis of C2 on C3

A computed tomography (CT) scan was subsequently completed and demonstrated a fracture of the odontoid process(Type II) and the right C2 pars interarticularis (Figure 2).

Magnetic resonance imaging (not shown) revealed significant spinal stenosis at C1-C2 with a posterior atlantodens interval (PADI) of 8mm. A PADI of less than 14mm indicates a high likelihood of neurologic deficit [5].

Final working diagnosis

Fracture of the C2 odontoid process and right pars interarticularis with C1-C2 spinal stenosis was the final diagnosis.

Case outcome

Considering the risk of spinal cord injury with an unstable atlantoaxial joint, orthopedics recommended an occiput to C5-C6 fusion with C1 laminectomy. The patient was hesitant about surgery and, in the meantime, wore a hard cervical orthosis. Pain management for this patient included cyclobenzaprine, tizanidine, indomethacin, and acetaminophen. The patient used a walker at baseline and engaged in outpatient physical therapy with a home exercise program. He experienced minimal symptom relief and functional gains, likely due to his baseline frailty and challenges with mobility due to the hard cervical orthosis. Unfortunately, four months after the fracture was diagnosed, the patient experienced an acute episode of atrial fibrillation that worsened his CHF. With an overall heavy burden of medical comorbidities, the patient chose to receive hospice care and therefore was managed non-operatively. The patient was alive with no further neurologic damage nearly one year after the fracture but continued to struggle with frailty. 

## Discussion

Both operative and non-operative management of Type II odontoid fractures have high rates of mortality and decreased functional status in elderly (age 65 and older) patients [3]. Studies suggest that mortality at one year is likely to be predicted more so by the patient’s baseline comorbidities rather than the C2 fracture itself [6,7]. Operative management of C2 fractures is increasingly the treatment of choice in the United States [8], although this trend is not global [4,9]. Several studies examining outcomes of surgical C2 fracture management in elderly patients demonstrated decreased rates of both short-term and long-term mortality compared to non-operative management [3,6,9,10]. In contrast, other studies found no significant difference in mortality at one year after diagnosis between patients treated operatively and non-operatively [7,11].

Operative management has a mortality benefit, likely due to improved upper cervical mobility that leads to better respiratory and swallowing function [9]. Type II odontoid fractures in the elderly have higher rates of non-union if managed without surgical intervention due to osteoporotic cortical bone without significant cancellous surface area. Furthermore, without proper blood supply, the fracture is less likely to heal [2,3]. As part of non-operative management, patients wear cervical orthoses or a halo vest, which are intended to immobilize the spine. However, because of the intentionally immobile nature of the orthoses and halo vests, patient function is compromised, leading to challenges in completing activities of daily living [12]. Of note, halo vests are sparingly used compared to orthoses due to high rates of respiratory complications, inadequate immobilization, and overall high rates of mortality [1,3]. 

Evidence for the management of vertebral fracture pain is poor. According to the literature, a stepwise approach is recommended and includes starting with a muscle relaxant and acetaminophen +/- nonsteroidal anti-inflammatory drugs and adding opioids if appropriate analgesia is not achieved [13].

After fracture union with operative or non-operative management, inpatient or outpatient rehabilitation is a cornerstone for functional gains. Interventions include patient education on neck pain mitigation, strengthening cervical and respiratory muscles, cervical range of motion exercises, balance exercises, and hand dexterity exercises in addition to neural mobilization, manual therapy, and gait training [14]. Early cervical mobilization following fracture is important for overall function and fitness and may be better facilitated by operative management [2,6]. In addition, gait aids such as a cane or walker are key tools to improve global mobility, especially if the patient suffered a concomitant injury after a fall [15].

## Conclusions

Non-operative management for a C2 odontoid fracture includes neck immobilization with cervical orthosis, physical therapy, pain management, and gait aids. In the elderly, operative management is recommended for Type II odontoid fractures. Morbidity and mortality are high in either treatment pathway. Surgical candidacy for fracture repair must be considered. Unfortunately, this patient’s overall frailty and past medical history of CHF made him unable to receive surgery. As a result, he enrolled in hospice care.
